# Supplementary material for: Electrochemical Adsorption on Pt Nanoparticles in Alkaline Solution Observed Using In Situ High Energy Resolution X-ray Absorption Spectroscopy
Source: Nanomaterials (Basel). 2019 Apr 20;9(4):642. doi: 10.3390/nano9040642 (PMC6523608; doi:10.3390/nano9040642)
Supplement: Supplementary file 1 [file nanomaterials-09-00642-s001.pdf]

nanomaterials

# Supplementary Materials

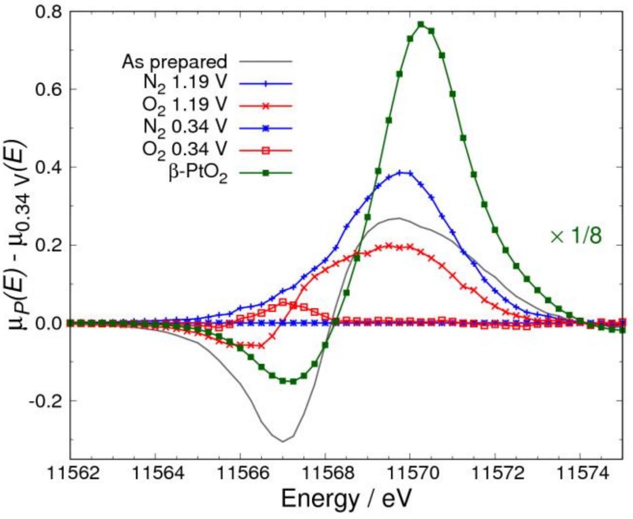


**Figure S1.** $\Delta\mu$ XANES spectra for the catalyst calculated by $\mu_p(\mathrm{E}) - \mu_{0.34\mathrm{V}}(\mathrm{E})$ where $\mu_{0.34\mathrm{V}}(\mathrm{E})$ is a spectrum in 1 M KOH saturated with $N_2$.

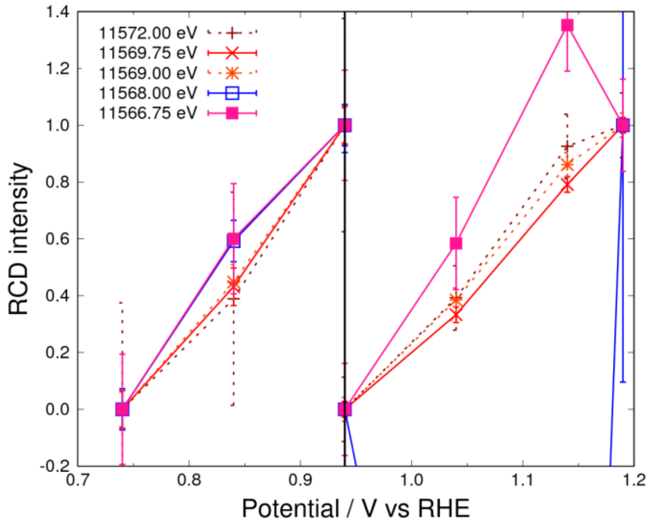


**Figure S2.** RCD curves for the controlled potential above 0.74 V in 1 M KOH saturated with $N_2$. The standard potentials are 0.74, 0.94 and 1.19 V. Circle and square curves represent the negative and two positive peaks of 11566.75 eV, 11567.5 eV and 11569.75 eV, respectively. The dashed lines are complement of the explanation for RCD.

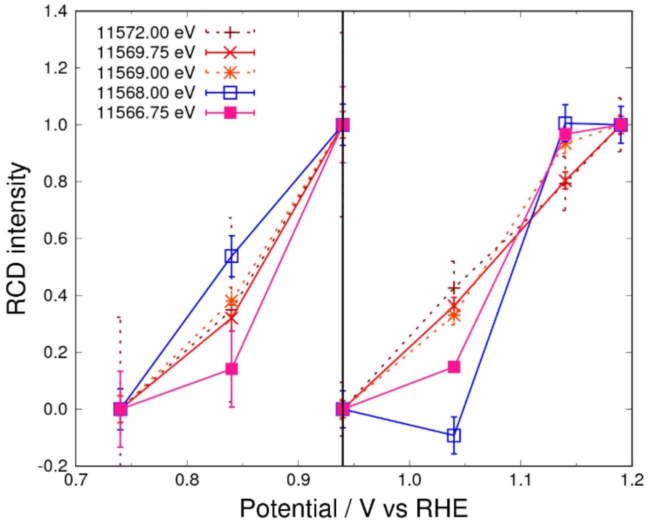


**Figure S3.** RCD curves for the controlled potential above 0.74 V in 1 M KOH saturated with $O_2$. The standard potentials are 0.74, 0.94 and 1.19 V. Circle and square curves represent the negative and two positive peaks of 11566.75 eV, 11567.5 eV and 11569.75 eV, respectively. The dashed lines are complement of the explanation for RCD.
